# Running together influences where you look

**DOI:** 10.1177/03010066241235112

**Published:** 2024-02-26

**Authors:** Eli Brenner, Marit Janssen, Nadia de Wit, Jeroen B.J. Smeets, David L. Mann, Andrea Ghiani

**Affiliations:** 1190 Vrije Universiteit Amsterdam, The Netherlands

**Keywords:** eye movements, depth, divided attention/resource competition, individual differences, locomotion, navigation/wayfinding

## Abstract

To read this article, you have to constantly direct your gaze at the words on the page. If you go for a run instead, your gaze will be less constrained, so many factors could influence where you look. We show that you are likely to spend less time looking at the path just in front of you when running alone than when running with someone else, presumably because the presence of the other runner makes foot placement more critical.

When walking on rough terrain foot placement is critical for each step, and people mostly look where they will soon place their foot. When walking on smooth surfaces foot placement is less critical, and half the time people look elsewhere (Matthis et al., 2018). People presumably weigh the need to look where they will place their foot with respect to other factors such as planning their further trajectory and checking out the environment. If so, the terrain is probably not the only factor that influences gaze. To see whether subtly changing the circumstances influences how people weigh the need to look where they will soon place their foot, we asked people to run a given path twice, once alone and once alongside another runner. When running together, there is less space on the path, and runners have to be prepared to adjust their upcoming foot placements to remain at an appropriate position with respect to the other runner should the latter move slightly differently than they anticipated. We therefore expected people to spend more time looking at the path just in front of them when running with someone else.

Twelve young adults took part in the experiment (approved by The Scientific and Ethical Review Board of the Faculty of Behaviour and Movement Sciences; file VCWE-2021-035). Their eye movements were recorded with Pupil Invisible eye trackers as they ran a 1.5 km trajectory twice, once alone and once together with someone else. When running together they were instructed to run side-by-side and to converse on the way. The paths were wide enough to comfortably do so (Figure 1A). On average, the trajectory took them 8.3 min when running alone and 9.2 min when running together. It presumably took them longer when running together both because they had to adjust to the speed of the slower runner and because they were conversing (Raffegeau et al., 2018).

**Figure 1.**
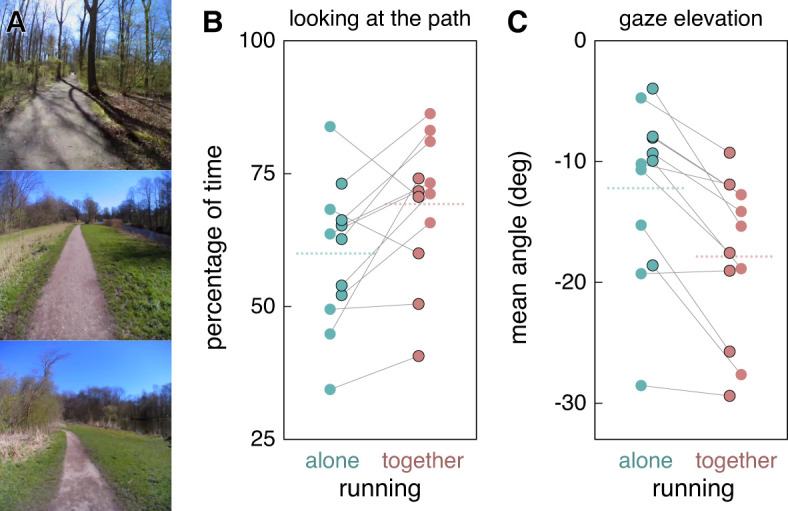
Images of parts of the path as captured by the eye tracker (A), how much of the time participants directed their gaze at the path (B), and participants’ average gaze elevation (C). Green and red symbols indicate individual participants’ values when running alone (green) or together (red). Thin lines connect individual participants’ values. The symbols have black borders if it was the participant's first run. Dotted lines show the mean values.

The proportion of time that gaze was on the path was examined by looking through the scene video with the gaze overlaid on the images, and judging for each image frame (30 Hz) whether gaze was or was not on the path. If participants stepped slightly off the path to avoid a puddle on the path or to pass people or dogs, we considered looking where they were soon to place their feet as looking at the path as long as they continued running, and doing so side by side if they were running together. Otherwise, that interval was excluded, as were intervals from when runners took a wrong turn until they were back on track, because their gaze was likely to be affected by the realization that they were no longer on the right path. As anticipated, on average participants looked at the path more of the time when running together than when running alone (Figure 1B; the 95% confidence interval of the mean difference of 9.2% was 6.4%).

We also determined the elevation of gaze as a measure of how nearby participants were looking. We determined the elevation of the head (at 200 Hz) from the output of the inertial measurement unit in the eye tracker, combining integrated rotation with the direction of gravitational acceleration. We determined the elevation of the eyes with respect to the head from the output of the eye tracker (also at 200 Hz, but excluding periods that were classified as blinks). As anticipated, we found that on average participants looked closer (lower gaze elevation) when running together (Figure 1C; the 95% confidence interval of the mean difference of 5.7° was 2.0°).

Not all participants behaved as we had anticipated. This is not surprising because many factors could make participants look differently during their two runs. For instance, half the participants first ran alone and the other half first ran together. On average, participants looked at the path more of the time the second time they ran (67% rather than 62% of the time). This might be because having already run the trajectory made it easier for them to plan where to go next, and had already given them the opportunity to check out the environment. Moreover, most participants only occasionally glimpsed at the other runner when running together, but one participant looked at her running partner more than 10% of the time. She is one of the two participants who looked at the path less frequently when running together.

We see considerable variability across participants, both in the amount of time spent looking at the path (Figure 1B) and in gaze elevation (Figure 1C). This is similar to what is found for walking up and down staircases: some people consistently look at most stairs while others look at very few (Ghiani et al., 2023). We interpret this as evidence that people differ in how they weigh the need to look where they will place their foot in relation to other factors, both when running and when ascending and descending staircases.

People appear to judge there to be more need to look where their feet will be placed when running alongside someone else. Running alongside someone else does not restrict foot placement to the same extent as does walking on rough terrain. Being closer to the edge of the path will constrain foot placement a bit, but there is no need to search extensively for suitable places to place one's foot, and the foot does not have to be placed as precisely at the selected position as when navigating rough terrain. On the other hand, when running alongside someone else one has to constantly consider having to adjust one's foot placement at short notice, because the other runner may occasionally not move precisely as anticipated. Irrespective of the precise contributions of these factors, our study demonstrates that subtly changing the circumstances can influence where people look.
